# Tribological and Antioxidation Synergistic Effect Study of Sulfonate-Modified Nano Calcium Carbonate

**DOI:** 10.1371/journal.pone.0062050

**Published:** 2013-05-03

**Authors:** He Zhongyi, Xiong Liping, Han Sheng, Chen Aixi, Qiu Jianwei, Fu Xisheng

**Affiliations:** 1 School of Basic Science, East China Jiaotong University, Nanchang, P.R. China; 2 Shanghai Institute of Technology, Shanghai, China; 3 PetroChina Lanzhou Lubricating Oil R & D Institute, Lanzhou, P.R. China; King's College London, United Kingdom

## Abstract

A middle base number sulphonate-modified nano calcium carbonate (SMC) with an average size of 35 nm was synthesized, and its tribological and antioxidation synergistic behaviors with ashless antioxidant N-phenyl-α-naphthylamine (T531) in hydrogenated oil (5Cst) were evaluated. The results demonstrate that adding this synethesized additive even at a low amount (<2.0 wt.%) can evidently improve its load-carrying capacity by 1.5 times and enhance its antiwear performance; in addition, the friction-reducing effect of additive in the high load was better than that in low load. The SMC have a good synergistic antioxidation effect with T531, which verifies the nano calcium carbonate compound was a kind of multifunctional and high-performance additive. The chemical composition of the rubbing surface which formed on the boundary film was analyzed by using scanning electron microscopy (SEM) and X-ray photoelectron spectroscopy (XPS). The results indicating that the excellent antiwear and load-carrying performance could be attributed to the forming of boundary lubrication film which composed of calcium carbonate, oxides, ferrites, sulphide and FeSO_4_, and so on. Its ability to increase oxidation free energy of base oil is the main reason for increasing its antioxidant collaboration property with ashless antioxidant T531.

## Introduction

Sulphonates are mainly used as metal detergents in lubricating oil, and they are mainly formed with calcium sulphonate, magnesium sulphonate, barium sulphonate, etc. They are applied in engine oil, antirust oil, gear oil, metal-working fluid, automotive grease, industrial grease and other fields according to different structures [1]∼[2]. Calcium sulfonates was introduced as detergents which can scavenge acidic contaminants from the lubricant originally. Some sulphonates (mainly sodium and calcium salts) have been found not only possess very good extreme pressure property in metal working fluid, but also possess very good solubility of nano carbonate particle in gel since the 1980's. Such an active film [3] that contains oxides and sulphides which come from additive the lubricating surface can prevent the contact with the substrate directly and plays an important role in extreme pressure behaviors. Recently, the development and research of inorganic nanoparticle as lubricant eliciting widespread interest, many nano-materials were added into lubricating oil/grease to improve load-carrying, anti-wear and friction-reduction properties [4]. Nanoparticles used as lubricant additive was considered an effective approach to upgrade lubricants due to their small size and physicochemical properties, which have been thoroughly investigated during the past decades [5]∼[7]. And these nanoparticles mainly focused on nano metal and metallic oxide, such as copper, nano calcium carbonate etc. [8], which modified with surfactant to improve solubility in base oil, and have poor oil-soluble stability. As to calcium carbonate, different crystalline forms possess different tribological property. For exmple, cubic CaCO_3_ that added in lithium grease can significantly improve it's antiwear, friction-reduction and load-carrying capacity [9].

With rapid industrial development, the modern equipment requires strengthen lubricating oil with oxidation-resistance property, such as high-temperature chain oil, synthetic aviation lubricant and other high-temperature lubricants. Currently, aromatic amine antioxidants, such as T531 and P, P-dioctyl diphenylamine, were mainly used as high-temperature antioxidant to suit the new requirements of modern machine. Many study results showed that adding oil-soluble metal salts in aromatic amine antioxidant is an effective way of to improve its high-temperature thermal oxidation stability. The metal salts are mainly alkali metal salts [10], which have no antiwear property, and earth metal salts has apparently not been investigated.

High temperature turbocharger test (TEOST 33C) was used according to the GF-5 specifications by the International Lubricant Standardization and Approval Committee (ILSAC) organization enforced in 2010,and test temperature is as high as 480°C, One pure antioxidant can not resist such a high temperature. The resulting stringent demand for two or more additives mix. According to this basis, adding oil-soluble metal salt into lubricant [3] is an effective way to improve the thermal oxidation stability at high temperature. Calcium sulphonate possess good oil-solution, when it was used as a kind of surfactant to modify CaCO_3_ nanopartices, it can improve solubility of CaCO_3_ in base oil. They offer anextremely attractive performance being as an additive for base oil, therefore, the study of CaCO_3_ nanoparticles is of great importance to us.

In this paper, alkyl benzenesulphonic acid was used as raw material to obtain middle base number calcium sulphonate, CaCO_3_ nanoparticles were prepared by the carbonation method. Being as additive in 5Cst, its tribological performances were investigated by using the four-ball tester, and the antioxidant property with T531 in 5Cst was evaluated by using pressure differential scanning calorimetry (PDSC). In addition, the lubricating mechanism was also discussed.

##  Additive Synthesis and Analysis Methods

### 2.1 Base oil and chemical reagents

A commercial hydrogenated oil product 5Cst, which ν100°C is 5.539 mm^2^·s−1, ν40°C is 31.60 mm^2^·s^−1^, flashpoint is 238°C, viscosity index is 110,made by Daqing Refinery Factory of China, was used as the lubricating oil without any further treatment. All chemical reagents (AP grade) are purchased from Shanghai Shen Bo Chemical co., LTD of China, the benzenesulphonic acid and HVI 150 base oil are made by Daqing Refinery Factory of China.

### 2.2 Additive synthesis

A four-flasks were installed with stirrer, thermometer, condenser evaporator, then added gasoline and methanol containing a certain amount of calcium oxide at 40°C, keep stirring, then added benzenesulphonic acid (industrial, with acid value 106 mg KOH/g), HVI150 base oil, heating to 120°C, leading gas containing carbon dioxide into the reaction chamber for 4 h to maintain alkalinity. After that removing the water and accelerant, when it cooled enough centrifuged it, the upper clear liquid was distilled under vacuum to obtain viscous bright red fluid. Calcium sulphonate which is middle base number, was abbreviated to SMC (The elemental analysis results: S% is 1.33, Ca% is 11.20, TBN is 282 mgKOH/g, ν100°C is 130.6). The peak at 881 cm−1 in the IR spectra of the samples is the absorption peaks of crystalline calcium carbonate.

### 2.3 Analysis methods

(1) Freeze-etching electron microscopy observation method: the sample was froze to liquid nitrogen temperature (negative 196°C), placed in a vacuum plating apparatus and then vacuumed. When the residual voltage reached to 0.004 Pa, the temperature drops to −150°C, the sample was fractured, after that, raise the temperature to −90°C, kept for 10 min in order to etching. After that the sample was withdrawn, washed with xylene, removed the film with copper net, and investigated it with electron microscopy [11]. The type of electron microscopy is HFZ-1 type.

(2) Specimens and testing apparatus: All test balls (φ 12.7 mm) which have been used in the test were made of GCr15 bearing steel with hardness HRc of 59–61. A microscope was used to determine the wear scar diameters (WSD) of the 3 lower balls with an accuracy ±0.01 mm. The Chinese standard test method GB3142-82, which is similar to ASTM D2783, was used to evaluate the maximum non-seizure loads, the rotation speed was conducted at 1450 rpm, keeping for 10 s, at room temperature. Friction and wear tests were conducted on a 4-ball test machine made in Ji nan Testing Machine Factory, China. The testing rotation speed was at 1450 rpm under different loads for 30 min. The worn scar was analysed by PHI-5702 type X-ray photoelectron spectroscope (XPS). And the additive was analysed by Thermo VG ESCA LAB 250 type X-ray photoelectron spectroscope (XPS). The radiation source was Mg K_α_ with pass energy of 29.35 eV, and the binding energy of C_1s_ (284.6 eV) was used as a standard value. A JSM-5600LV type scanning electron microscope (SEM) was also used to study the rubbed surface morphology.

## Results and Discussion

### 3.1 Freeze-etching electron microscopy of the product

**Figure 1:**
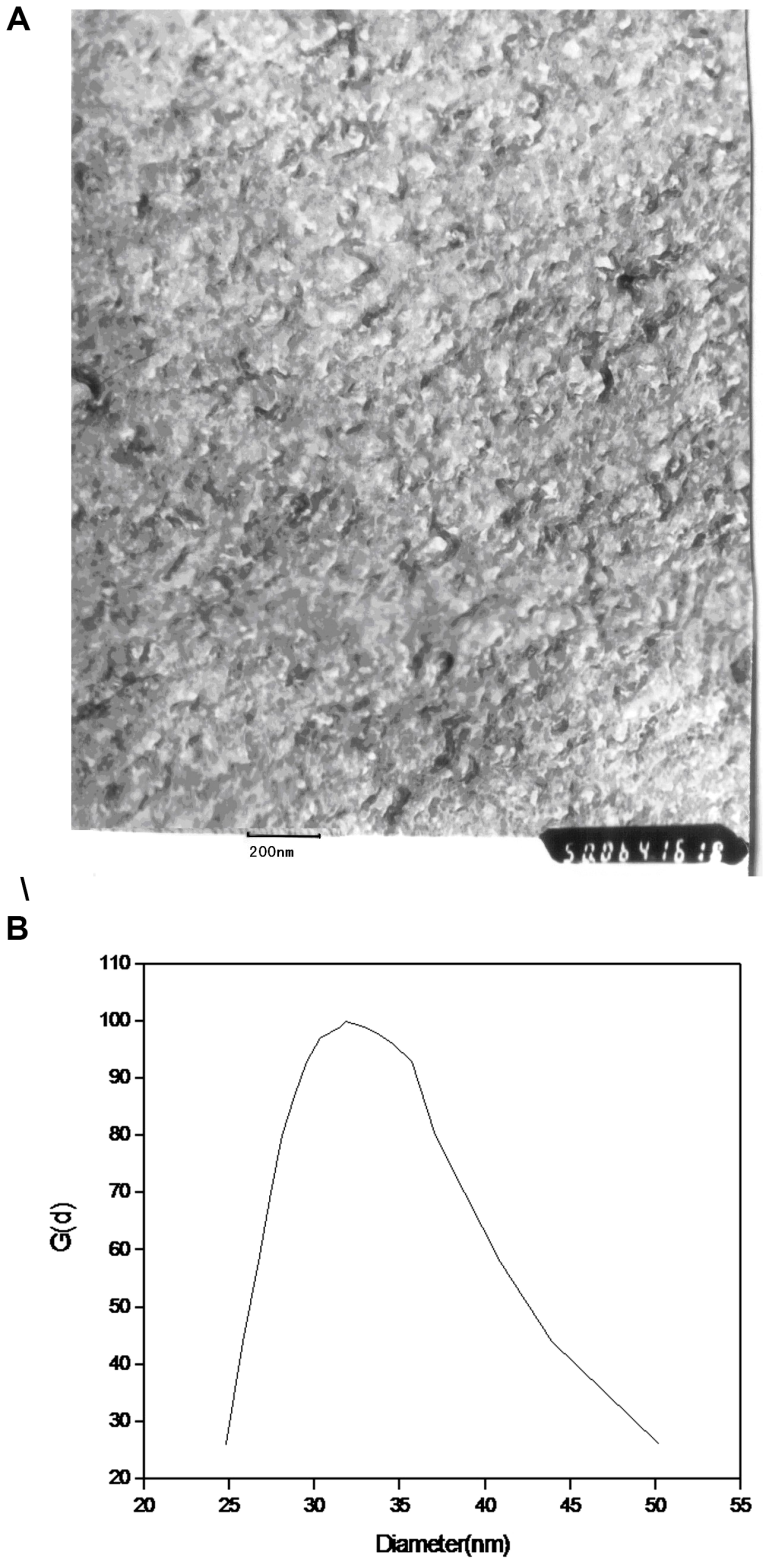
Freeze-etching electron microscopy (A) and Granularity (B) of SMC.

The enlarged 100,000 times freeze-etching electron microscopy of synthesized nano calcium carbonate is shown in Figure 1. The SMC shown like flake shape, instead of the ball shape, its size range from 20 to 50 nm, and the weight average particle size is about 35 nm. It is crystallization calcium carbonate, and it has a different from traditional calcium sulphonate product. As a detergent agent, the particle size of CaCO_3_ should be less than 80 nm [12], otherwise it will cause product to turbid, and the poor colloid stability will affect its using performance. Some research shown that the right particle size of load micelle should be under 20 nm, and uniformly distributed.

Its grain size analysis used the Zetaplus/90 plus type zeta laser particle size analyser, images has been also shown in Figure 1. It can be seen that the average size of SMC is about 35 nm.

### 3.2 Tribological properties

The maximum non-seizure load (*P*_B_ value) results of base oil and containing different concentration additive at room temperature were shown in Table 1. The *P_B_* value of SMC is 1.5 times of base oil at 2.0 wt %. With the additive concentration increasing, the *P*_B_ value increases accordingly, but high concentration does not corresponding to high P*_B_* value when concentration over 2.0 wt%.

**Table 1:** P*_B_* value of additives in base oil.

Additive Concentration	0.0	0.5	1.0	2.0	3.0	5.0
P_B_(N)	392.3	441.3	539.0	617.8	559.0	588.4

**Figure 2:**
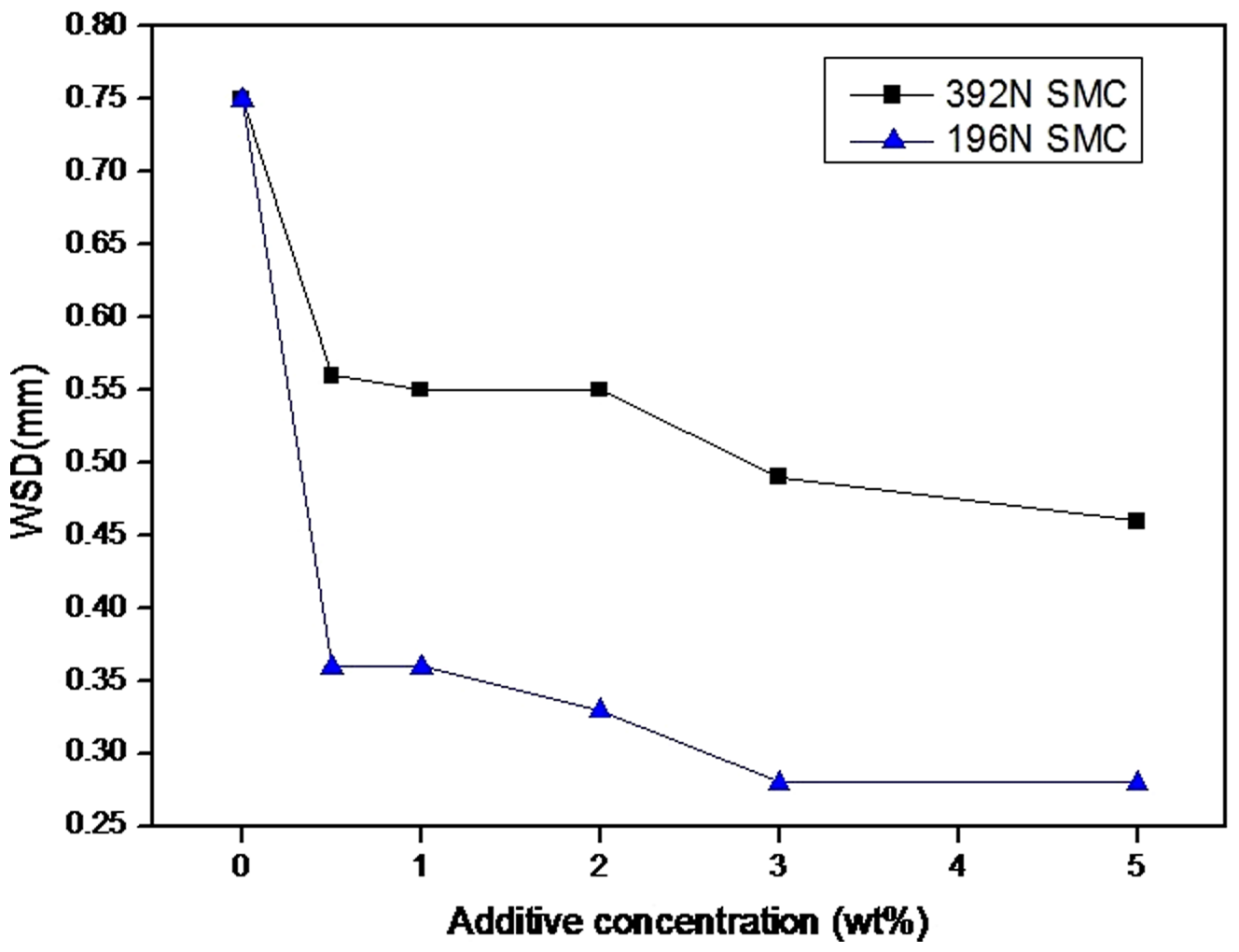
Wear scar diameter of additives.

Figure 2 shows the wear scar diameter (WSD) at 392N and 196N. It can be seen that in the presence of SMC, the WSD decrease compared to those of the base oil. The additive possesses better antiwear property along with additives concentration increasing, meanwhile, it become smoother when additive concentrations increasing over 0.5wt%. And it possesses better antiwear property at lower load than at higher load.

**Figure 3:**
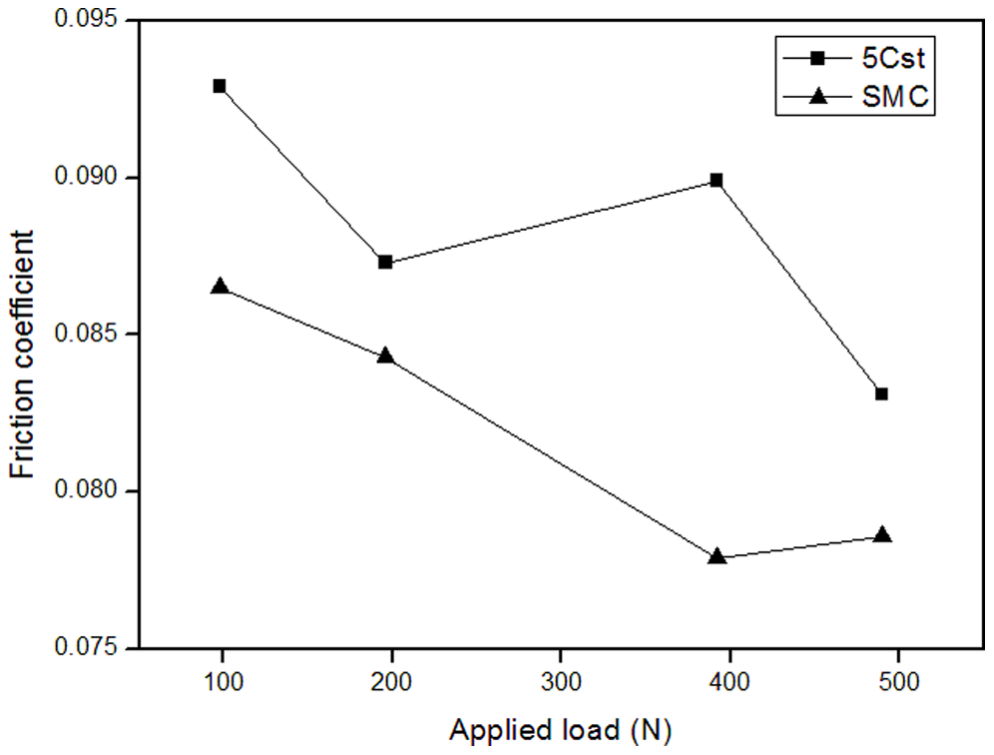
Effect of load on friction coefficient.

Figure 3 was shown the relation between the different load and the friction coefficient, and that of the oil containing 2.0 wt% SMC was investigated to reference to that of the base oil. The friction coefficient shows low value at 392N after addition of SMC, the friction-reducing effect of additive in the high load was better than in the low load.

### 3.3Antioxidant properties

The oxidation induction period (t_on_) of PDSC was used to study antioxidation property of SMC and T531. The oxidation process of base oil is an exothermic reaction, when the base oil becoming oxidized, it will appear apparent exothermic peak in the PDSC curves under heating conditions with oxygen. The lower t_on_ of base oil, means the worse oxidation stability. The results of t_on_ at different temperatures have been tabulated Table 2.

**Table 2:** t_on_ under different temperature.

Temperature		145°C	150°C	155°C	160°C	170°C	180°C	190°C
t_on_/min	5cst	42.3	28.3	19.2	13.1			
	0.25% T531/5cst				>120	60.9	24.7	10.8
	0.025wt% SMC+0.25wt%T531					76.4	33.5	12.2

It can be seen from Table 3 that with the temperature rising, the t_on_ of base oil is significantly reduced, which means that higher temperature helps proceeding oxidation reaction.

**Table 3:** Oxidation activation energy of oil samples.

oil sample	5cst	0.23% T531/ 5cst	0.023wt% SMC + 0.23wt%T531
Ea (kJ/mol)	117.015	147.440	156.707

The oxidation reaction of base oil with aromatic amine antioxidant is a free radical reaction [13], and the activation energy of the oxidation reaction can be calculated by using the Arrhenius formula according to PDSC test results. Because the chemical activation energy measure describes the difficulty of chemistry reaction. And in general, the smaller activation energy, the easier to proceeding the reaction. So the chemical activation energy response to the difficulty of reaction degree, it can be used to study its oxidation mechanism.

**Figure 4:**
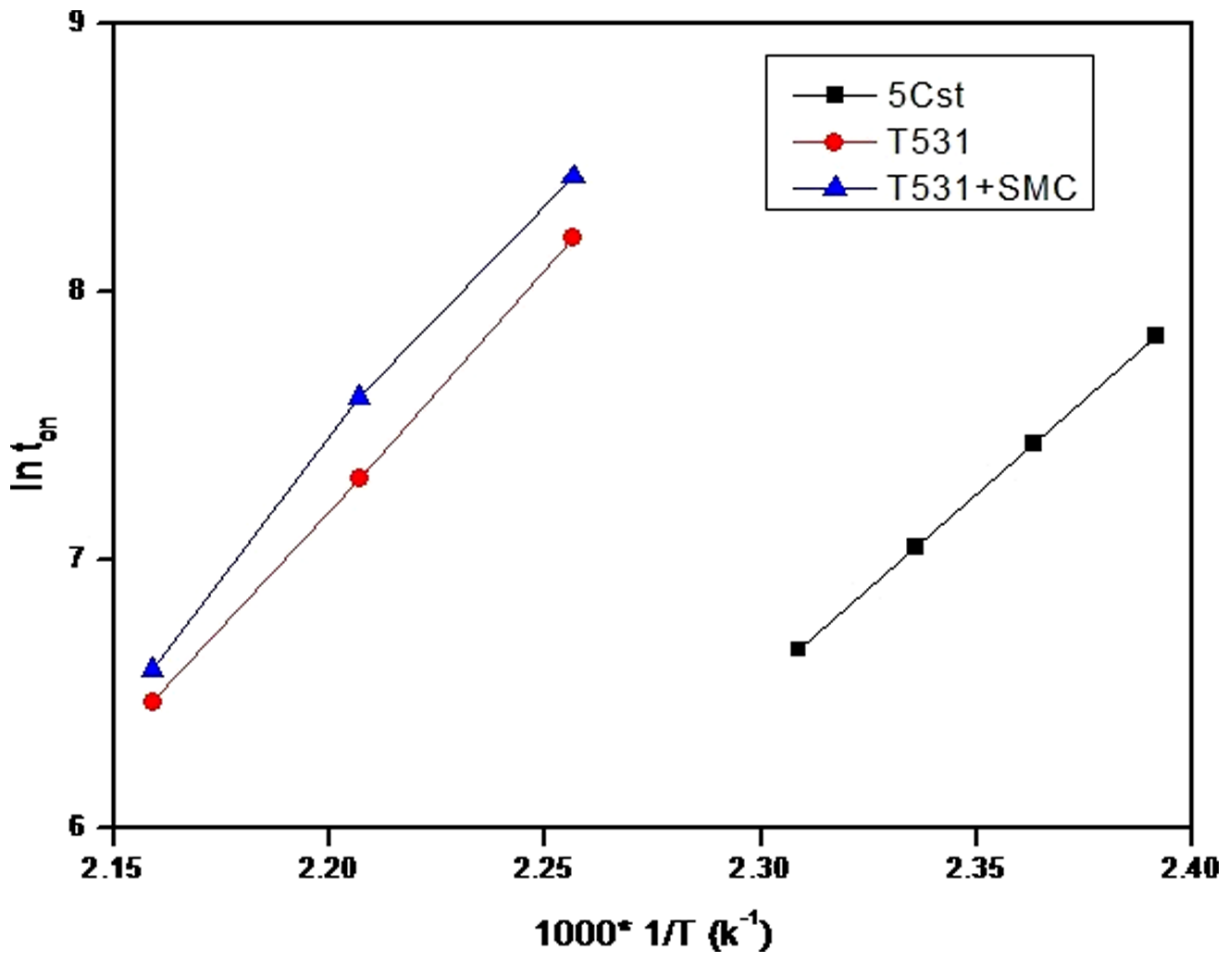
ln t_on_∼1/T curve at different temperatures.

According to Arrhenius equation: lnk = −Ea/RT+C, and k is the oxidation rate constant, and k is inversely proportional with the reaction time in the initial stages of oxidation, therefore, then it can deduce the following equation [14]: ln t_on_ = Ea/RT−C′. Then we used lnt_on_ as the y-axis, the 1/T as the x-axis, we can obtained a straight line in theory, the slope equal to Ea/R. The activation energy Ea of oxidation reaction have been calculated, the result was shown in figure 4.

In Figure 4, it can be seen that the resulting curves were essentially straight lines, which means that the oxidation curves of base oil and containing additive base oil are in accordance with the Arrhenius formula, and that the activation energy calculated according to this method is feasible. The calculated values of oxidation activation energy of the oil samples are shown in table 3.

The activation energy data shows that the synthesized calcium sulfonate can improve the activation energy of oxidation reaction very well, and they shall be able to improve the antioxidant effect of base oil effectively.

### 3.4 The worn surface analysis of steel ball surface

**Figure 5:**
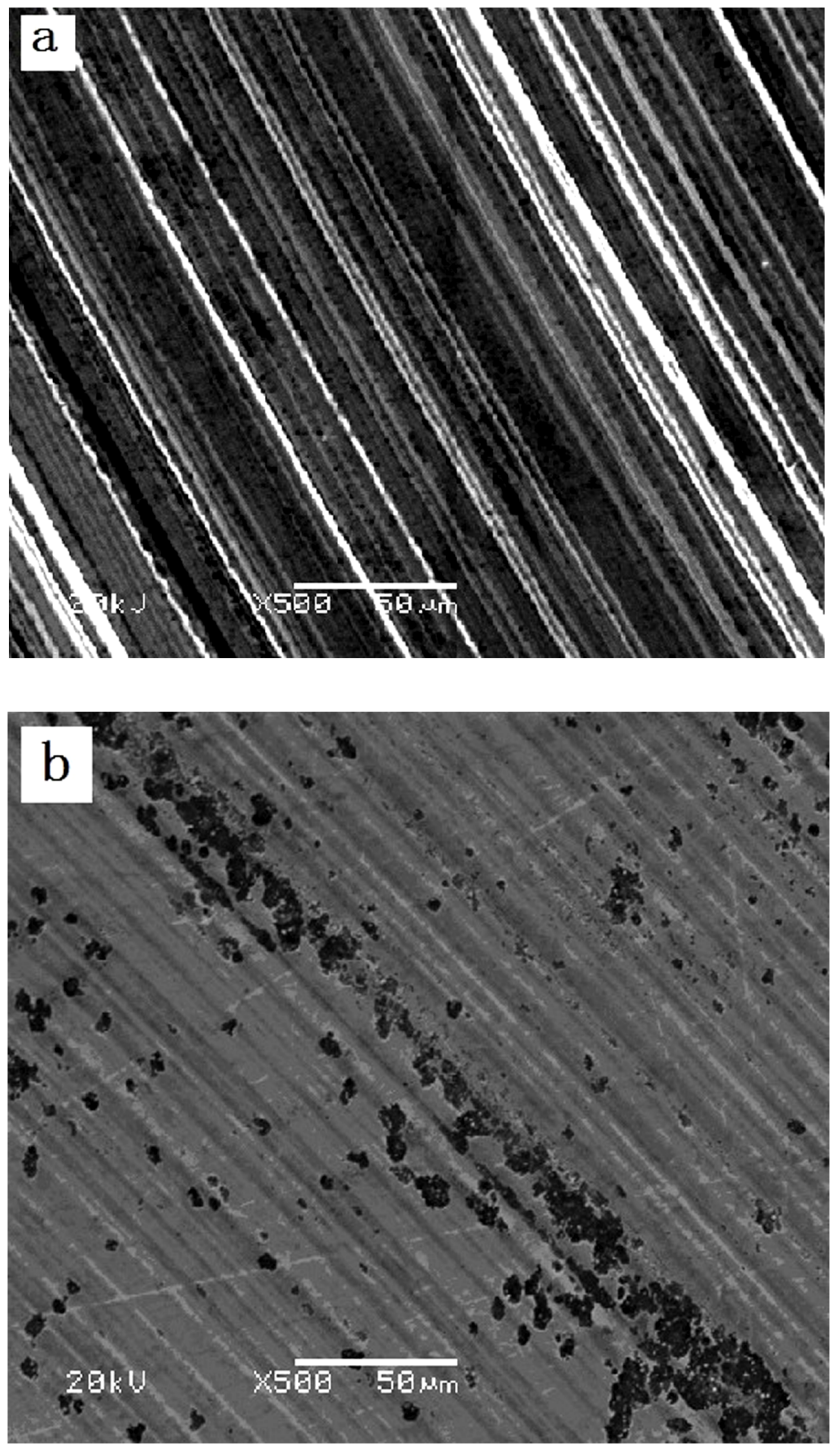
SEM morphologies of wear scar of steel balls lubricated by a 5Cst(a) and b 1.0 wt% SMC/5Cst(b) at 392N for 30 min.

In order to understand the boundary lubricating mechanism, the worn surface of steel ball was studied by SEM. Figure 5 gives the typical SEM images of the worn steel ball surface lubricated by 5Cst and the 5Cst containing 1.0 wt% SMC at 392 N for 30 min. It can be seen that the surface lubricated by 5Cst alone is rough and shows signs of severe scuffing (Figure 5a), taking on grain abrasion characteristic. On the other hand, scuffing on the surface of the tested steel ball lubricated by 5Cst containing 1.0 wt% SMC was significantly inhibited (Figure 5b). Moreover, the wear scar of the steel ball lubricated by 5Cst containing 1.0 wt% SMC is much smaller and smoother than that lubricated by 5Cst alone. Therefore, the results further testify that SMC has good anti-wear property.

Table 4 was atoms concentration of worn surface lubricated by 5Cst and 5Cst containing 1.0 wt % SMC at 392 N for 30 min. It can be seen that the elements of C, O, Cr and Fe were present on the worn scar surface lubricated only by 5Cst. The elements of Ca and S were present on the worn scar surface lubricated by 5Cst containing 1.0 wt % SMC, and its atomic concentration of Ca was 0.36%, S was 0.18%. This fact shows that Ca in CaCO_3_ nanoparticle, S in calcium sulfonate were deposited on the worn steel surface in the process of friction. The presence of Ca gives strong evidence that a lubricate film must be formed and probably contains CaCO_3_ nanoparticle and/or calcium oxide, which can prevent the steel-to-steel direct contact.

**Table 4:** Atoms concentration of worn surface.

Element		C	O	S	Ca	Cr	Fe
Atomic%	5cst	77.52	5.63	0.0	0.0	0.31	16.54
	1.0% SMC/5cst	34.97	0.71	0.18	0.36	0.87	62.90

**Figure 6:**
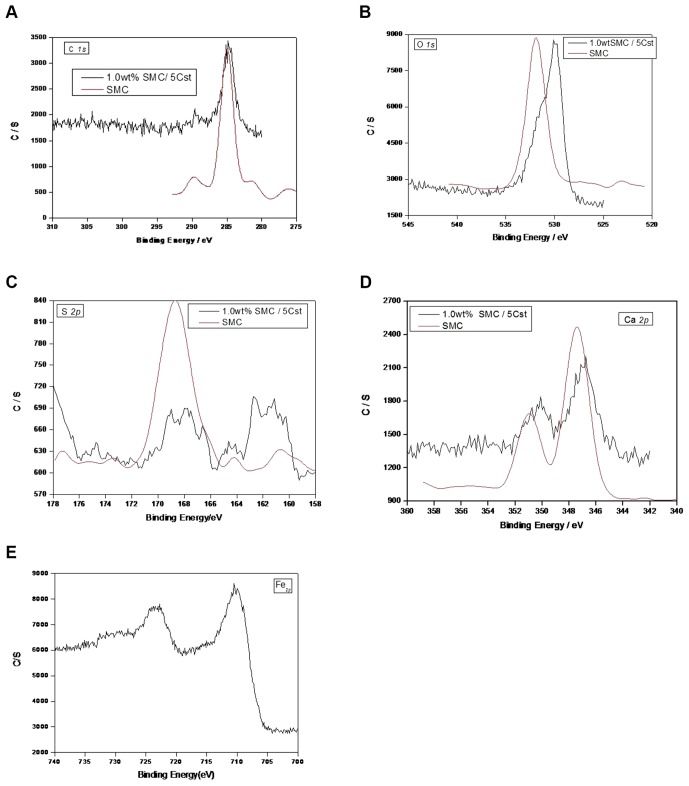
XPS spectra of the characteristic elements of SMC and s on worn surface lubricated by 1.0 wt% SMC/5Cst at 392 N for 30 min.

In order to understand the tribological mechanism of the SMC in the lubricating oil, the additive-derived elements: carbon, oxygen, sulfur, and calcium were detected by XPS analysis, and these elements which analysed of worn surface lubricated with SMC at 392 N for 30 min were also detected by XPS. The results were shown in figure 6.

The binding energy peak of C*_1s_* appears at 289.8 eV, which is identified as C in carbonate [15]. Besides, the binding energies of C*_1s_* at 284.9 eV directly correspond to C-H and C-O, which existing in the additive and base oil, and it means that the base oil and additive were adsorbed on the metal surface. For worn steel ball surface, the binding energy peak of C*_1s_* appears at 284.9 eV, which is identified as C in C-H and C-O the manuscript presented in an intelligible fashion and written in standard English. The weak peak at high binding energy of 289.8 eV is associated with carbonate [16].

For additive SMC, the peak of O*_1s_* appears at 531.9 eV, corresponding to calcium sulfonate and calcium carbonate, and the peak at 529.0 eV, corresponding to calcium oxide, hasn't appear, it means that calcium oxide perhaps wrapped in the calcium carbonate, or maybe SMC does not cintain calcium oxide. For the tested steel ball worn surface, the O*_1s_* peak appears at 529.6 eV can attribute to oxygen in iron oxide and calcium oxide.

For additive SMC, the peak of S*_2p_* appears at 168.7 eV, corresponding to sulfonate. But the peaks have changed to 168.8 eV, 167.8 eV, 161.4 eV and 159.5 eV on the worn surface after lubricating process, which corresponding to FeSO_4_, FeS respectively. The binding energy of S*_2p_* is 168.8 eV, which corresponds to FeSO_4_ [17], and it means that S element of sulfonate was adsorbed on the steel balls surface mainly in the form of ferrous sulphate after tribological process.

For SMC, the main peaks of Ca*_2p_* appear at 351.0 eV and 347.3 eV were attributed to calcium in CaCO_3_ and CaO respectively. Also Ca*_2p_* at 347.3 eV and O*_ls_* at 529.6 eV are identified as calcium oxide. For the steel ball worn surface, the main peak of Ca*_2p_* appears at 346.7 eV is attributed to calcium in CaO.

The Fe*_2p_* peak appears around 710.6 eV is attributed to the generation of iron oxide. And this conclusion was also supported by the binding energies of O*_1s_* being around 530 eV. Another peak appeared at 723.5 eV was attributed to Fe in FeSO_4_.

The XPS analysis of the outside worn surface lubricated by blank base oil only presents C, O, and Fe elements peaks [18], hadn't detected Ca and S elements. The XPS analysis mentioned above indicated that thin boundary lubrication film have been formed and this film contained CaCO_3_, CaO, iron oxide and FeSO_4_,FeS etc., and some organic compounds coming from 5Cst itself.

According to the above XPS analysis results, discussion on the lubrication mechanism of the sulfonate-modified calcium carbonate nanoparticals as additives in 5 Cst are as followings.

First, the calcium sulfonate was vertically adsorbed on steel ball surface according to “preferential orientation” adsorption mechanism [19], which taking part in a competitive adsorption with base oil during lubricating process. And the sulfonate-modified CaCO_3_ nanoparticles contained in the 5Cst are trapped inside the contact and fill in the gap between the rubbing surfaces, the additive facilitated reduction of friction and wear. The tribological action between metals will produce high temperature, the alkyl of calcium sulfonate was easily sheared under boundary lubrication conditions. The nanoparticles of CaCO_3_ can deposite on the shearing surface because of the high surface energy of the fresh worn surface, and the complicated tribochemical reaction that had occurred in reaction system. The additive molecules have been decomposed [20], the sulphonic acid group reacts with the metal surface forming sulphate, sulphide and ferrite film, the inorganic film has high hardness, which made the tribological surface film acquired higher load-carrying capacity. And the calcium carbonate reacts with iron oxide and fresh iron on the worn surface to generate calcium oxide and ferrite calcium. The tribochemical reaction film [21] is composed of absorbed organic materials which comes from additives or 5Cst itself, the generating CaO, iron oxide, sulphate, sulphide etc.. Besides, the tribochemical compounds which act as solid lubricants during the friction process, and tribochemical reaction products on the worn surface improve the tribological properties of 5Cst.

## Conclusion

From the above results, the following conclusion can be drawn:

(1). The synthetic sulphonate-modified calcium carbonate can improve the extreme pressure performances of base oil. The extreme pressure performance increases with the increase of additive concentration. The additive has a good antiwear property, and the friction-reducing effect of additive at the high load was much stronger than that at low load.

(2). The oxidation stability of additive is poor, but it can improve the antioxidation property future of the base oil by increasing the activation energy of oil sample during oxidation reaction process, when the additive is mixed with the antioxidant T531.

(3). As a lubricating oil additive, the sulphonate-modified nano calcium carbonate occured tribochemical reaction with steel ball in the friction process. The sulphonate formed inorganic salts such as oxides, FeSO_4_ and sulphide, and the carbonate particle formed calcium oxide and ferrites, which formed a complex boundary film on the surface. The mixed reaction boundary film adsorbed on the worn steel ball surfaces can improve the tribological properties of the base oil.
